# More Evidence on the Effects of Deworming: What Lessons Can We Learn?

**DOI:** 10.4269/ajtmh.17-0161

**Published:** 2017-06-07

**Authors:** Kevin Croke, Eric Hsu, Michael Kremer

**Affiliations:** 1 Development Economics Group, World Bank, Washington, District of Columbia; 2 Department of Economics, University of California, Berkeley, Berkeley, California; 3 Department of Economics, Harvard University, Cambridge, Massachusetts

In this month's issue, Liu and others present new evidence on the effects of deworming children from a cluster-randomized trial conducted in rural China.^1^ This was a useful, well-conducted study. The study found that a program in which school-age children were given deworming pills in school to take at home reduced infection prevalence modestly (end line prevalence of any worm infection was 31.4% in the control group and 27.7% in the treatment group). However, the authors could not reject the hypothesis that the program had no effect on nutrition, cognition, or school performance.

As Liu and others emphasized, their results should be interpreted in light of the context they examined. The authors noted that in their setting, baseline prevalence of soil-transmitted helminths (STH) was low (31% for *Ascaris*, 23–24% for *Trichuris*, and 1% for hookworm), infections were “light intensity,” and even within the light-intensity group, measured egg counts were low. For example, mean infection intensity among children infected with *Ascaris* was less than 1,000 eggs per gram (epg; up to 4,999 epg is a light infection) and infection intensity for *Trichuris* was less than 70 epg (up to 1,000 epg is a light infection).^2^ The authors are to be commended for reporting prevalence and intensity for all three STH species, which greatly aids the interpretability of their results.

Compliance with the deworming treatment was low, presumably because the study differed from most school-based deworming programs, and in particular from the standard World Health Organization (WHO) protocol; in accordance with Chinese regulations preventing children from taking the drugs at school, students were given pills to consume at home. Only 52% of participants reported taking all the recommended pills (75.6% reported taking at least half of the recommended dose of albendazole [200 mg] in both rounds of treatment). Compliance was self-reported, so any social desirability bias by respondents would mean that true compliance was lower than 52%. The fact that the end line difference in prevalence between the treatment and comparison group was only 3.7% points could potentially be explained by a combination of high rates of reinfection and this relatively low rate of compliance.

It is useful to first consider the point estimates of effects in the Liu study and then consider issues of hypothesis testing and statistical power. The estimated effects in the Liu study were largely consistent with the results of a meta-analysis recently conducted by Croke and others^3^ on the impact of mass drug administration (MDA) on weight gain. The Croke analysis found that, in the environments where WHO recommends deworming, school-based MDA was effective in increasing weight, and indeed that it was very cost-effective relative to school feeding (school feeding was an example of a nutrition program that targeted similar populations for which, based on a review of randomized-controlled trials, the authors were able to identify a cost-effectiveness analysis).^4^ Although too few studies have been conducted to draw strong conclusions about how effects vary with prevalence, point estimates are consistent with the commonsense view that effects are smaller in lower prevalence environments. Among studies in environments with greater than 50% infection prevalence in the Croke study, the average weight gain estimated in a random effects meta-analysis was 0.18 kg (95% confidence interval [CI] 0.07, 0.29). In contrast, the average effect in settings with less than 50% infection prevalence was 0.06 kg (95% CI −0.13, 0.25).^3^ A separate meta-analysis, by Taylor-Robinson and others,^5^ also estimated an average weight gain of 0.06 kg in under 50% infection prevalence settings.

In this lower prevalence sample, the Croke study estimated that the standard deviation of true effects was 0.25 kg. Using the method of Higgins and others,^6^ this implies that approximately 95% of places are expected to have a true effect in the range −0.59 to 0.71 kg. To put the outcomes in comparable terms, the weight gain estimate from the Liu study expressed in kilograms (rather than weight-for-age z score) was 0.03 (95% CI −0.25, 0.32). This clearly was well within the expected range.

It is also unsurprising that the point estimates in the Liu study were lower than the estimated mean effects from this meta-analysis, since reported compliance was only 52% and infection prevalence at end line was only 12% lower in the treatment group. Although not consistently reported, compliance was typically 80–100% in other trials incorporated in the meta-analysis. Assuming that treatment effects were proportional to compliance and compliance was 0.52 implies that with full compliance, the estimated effect in the Liu study would have been (1/0.52) × 0.03 kg = 0.06 kg, close to the average estimated effect found by Croke and others in studies with less than 50% prevalence.

Thus, while the results of the Liu study were consistent with the Croke analysis and with the hypothesis that MDA is cost-effective, the confidence intervals were wide enough to also encompass zero effect. The study was larger and better powered than many earlier trials, but as discussed later, power is inherently limited due to low prevalence and low compliance. Moreover, while the cluster-randomized nature of the trial was necessary to pick up potential epidemiological spillovers from treatment and thus to accurately measure impact, it did limit statistical power.

Statistical power is limited in low-prevalence settings, since any effect on infected children will be averaged together with effects on uninfected children, who cannot benefit directly from treatment. The authors state that among the 10 outcomes of interest, STH prevalence was the outcome that required the largest sample to detect a 0.25 standardized effect, and that they therefore powered the study based on this outcome.

It is, however, unclear what the minimum detectable effects (MDEs) were for the main outcomes other than STH prevalence. We do not know the MDEs for individual-level outcomes besides STH infection, so we cannot estimate the smallest detectable treatment-on-the-treated (TOT) effect and therefore cannot evaluate whether the smallest detectable TOT effect for an outcome was reasonable, given results from existing studies on deworming. More importantly, knowing the MDEs for each outcome would also allow us to compare MDEs with an effect size at which deworming would be cost-effective.

Compliance also affects power to detect effects on those who were actually treated. Liu and others did not state whether their power calculations adjusted for expected noncompliance. This required adjustment would be large: since only 52% of the sample reported taking all required doses of albendazole, the sample would have to be up to (1/0.52)^2^ = 3.7 times larger than when assuming full compliance.

We have focused on the weight outcome for comparability to the Croke meta-analysis, but effects on other variables are typically not estimated precisely enough to rule out either zero effect or effects that would make MDA cost-effective. In general, there is no clear pattern to the estimated effects, with both positive and negative insignificant point estimates, but it is worth noting that two estimated effects had *P* values near 0.1. Working memory improved by 0.51 (*P* = 0.093) and treatment group children were 23% less likely to be underweight (adjusted odds ratio 0.77, *P* = 0.113).

Note that the limited statistical power was not due to any fault of the authors, who took a number of sensible steps to improve power, such as matched pairs randomization, controls for pre-treatment covariates to improve precision, and addition of 12 extra clusters to account for attrition. Rather, it is inherent in examining settings with limited prevalence and intensity, particularly given the Chinese regulations that limited compliance.

In sum, we would not interpret the results of the Liu study as suggesting a reason to abandon the WHO's recommendation for MDA in endemic areas. Indeed, one could interpret the results as suggesting the importance of implementing MDA according to the standard WHO approach, in which children consume the pills in school. As even skeptics of MDA acknowledge, treatment of infected children is warranted, and MDA is the most cost-effective way to treat heavily infected children, given the low cost of MDA and the high cost of diagnosis and treatment.

In our view, debates over whether to conduct MDA are misplaced. The appropriate question is under what circumstances the statistical expectation of benefits of MDA (taking into account uncertainty about those benefits) exceeds its cost. We would not endorse MDA in a population with no worm infections. Even those most skeptical of MDA endorse treatment of those known to be infected, and thus would presumably support MDA in a population with 100% prevalence and the relatively high intensity of infection that typically accompanies high prevalence. Yet the expected benefits of deworming are clearly continuous across prevalence and intensity, so simple logic suggests that MDA is inappropriate in populations with low enough prevalence and appropriate in populations with high enough prevalence. The relevant question is, at what threshold is MDA justified? The WHO has made the judgement call that 20% is an appropriate threshold. That seems reasonable, but one could legitimately ask if the threshold should be higher or lower, or take into account intensity as well as prevalence, or vary by STH species or other factors. From a Bayesian decision theory point of view, the choice of threshold should be informed by comparing the expected benefits of treatment versus the costs. However, the expected benefits depend on relationships that are not currently well understood, including the extent to which treatment benefit is driven by children with medium-to-high intensity infections, the relationship between prevalence of any infection and prevalence of medium-to-high intensity infections, as well as dollar valuations for the impacts of deworming. Since statistical power to detect effects in low-prevalence populations is limited, progress in this area will be difficult, and will need to rely on modeling as well as trials.
